# Stability Evaluation of Different Oblique Lumbar Interbody Fusion Constructs in Normal and Osteoporotic Condition – A Finite Element Based Study

**DOI:** 10.3389/fbioe.2021.749914

**Published:** 2021-11-05

**Authors:** Ferenc Bereczki, Mate Turbucz, Rita Kiss, Peter Endre Eltes, Aron Lazary

**Affiliations:** ^1^ In Silico Biomechanics Laboratory, National Center for Spinal Disorders, Budapest, Hungary; ^2^ School of PhD Studies, Semmelweis University, Budapest, Hungary; ^3^ Department of Mechatronics, Optics and Mechanical Engineering Informatics, Budapest University of Technology and Economics, Budapest, Hungary; ^4^ Department of Spine Surgery, Semmelweis University, Budapest, Hungary

**Keywords:** degenerative disc disease, spine surgery, finite element analysis, osteoporosis, oblique lateral interbody fusion, stand-alone

## Abstract

**Introduction:** In developed countries, the age structure of the population is currently undergoing an upward shift, resulting a decrease in general bone quality and surgical durability. Over the past decade, oblique lumbar interbody fusion (OLIF) has been globally accepted as a minimally invasive surgical technique. There are several stabilization options available for OLIF cage fixation such as self-anchored stand-alone (SSA), lateral plate-screw (LPS), and bilateral pedicle screw (BPS) systems. The constructs’ stability are crucial for the immediate and long-term success of the surgery. The aim of this study is to investigate the biomechanical effect of the aforementioned constructs, using finite element analysis with different bone qualities (osteoporotic and normal).

**Method:** A bi-segmental (L2–L4) finite element (FE) model was created, using a CT scan of a 24-year-old healthy male. After the FE model validation, CAD geometries of the implants were inserted into the L3–L4 motion segment during a virtual surgery. For the simulations, a 150 N follower load was applied on the models, then 10 Nm of torque was used in six general directions (flexion, extension, right/left bending, and right/left rotation), with different bone material properties.

**Results:** The smallest segmental (L3–L4) ROM (range of motion) was observed in the BPS system, except for right bending. Osteoporosis increased ROMs in all constructs, especially in the LPS system (right bending increase: 140.26%). Osteoporosis also increased the caudal displacement of the implanted cage in all models (healthy bone: 0.06 ± 0.03 mm, osteoporosis: 0.106 ± 0.07 mm), particularly with right bending, where the displacement doubled in SSA and LPS constructs. The displacement of the screws inside the L4 vertebra increased by 59% on average (59.33 ± 21.53%) due to osteoporosis (100% in LPS, rotation). BPS-L4 screw displacements were the least affected by osteoporosis.

**Conclusions:** The investigated constructs provide different levels of stability to the spine depending on the quality of the bone, which can affect the outcome of the surgery. In our model, the BPS system was found to be the most stable construct in osteoporosis. The presented model, after further development, has the potential to help the surgeon in planning a particular spinal surgery by adjusting the stabilization type to the patient’s bone quality.

## Introduction

Lumbar interbody fusion (LIF) is a gold-standard surgical treatment option for a range of spinal disorders, including degenerative pathologies, infection, trauma, and neoplasia (Mobbs et al., 2015; Resnick et al., 2005). LIF can be achieved via different approaches and techniques, each with its own unique instruments, implants (exp. cages), advantages, disadvantages, indications, and limitations. The age structure of the global population is currently undergoing an upward shift due to decreasing fertility rates and increasing life expectancy (Fuster, 2017), resulting in the changing epidemiology of diseases and spinal disorders (Fehlings et al., 2015). Advancement in minimally invasive spinal fusion technology (Yue et al., 2010) can provide an answer for the challenges posed by the ageing population (Shamji et al., 2015). The minimally invasive anterior approach to the lumbar spine through retroperitoneal access was first described by Mayer (1997). Silvestre et al. (2012) used Mayer’s minimally invasive retroperitoneal anterior approach for LIF, and it was referred to as oblique lumbar interbody fusion (OLIF). The OLIF technique is widely accepted (Mobbs et al., 2015; Phan et al., 2016) and provides, from the patient’s left side, a safe access corridor from L2 to L5 vertebra between the psoas and the aorta. Through the corridor, the surgeon can resect the disc, remove the cartilage endplate, insert a large intervertebral cage, and achieve the goal of intervertebral fusion and indirect decompression (Mehren et al., 2016) by keeping the lumbosacral plexus safe (Phan et al., 2016; Mehren et al., 2016; Chung et al., 2017). During the OLIF procedure, different additional fixation methods can be applied, and there is no consensus about the indication for choosing a particular type. Self-anchored stand-alone (SSA) OLIF cages contain a screw fixation part besides the intervertebral spacer. Lateral plate-screw (LPS) fixation has a longer history in spinal trauma, but a new plate design has recently emerged, dedicated for OLIF. Percutaneous bilateral pedicle screw (BPS) fixation can be used after turning the patient to prone position, which, in general, increases the operation time (Li et al., 2020) and the invasiveness by the posterior incisions used to insert the pedicle screws. For experienced spine surgeons, there is no difference in the complexity of the 3 procedures. Several other fixation methods and a combination of these have been reported considering their technical specifications. However, only a few studies have investigated the biomechanical characteristics of OLIF with various fixation options (Hah and Kang, 2019), especially focusing on the effect of osteoporosis, which is widely present in the ageing population (Fehlings et al., 2015). Biomechanical characteristics of the different OLIF constructs can significantly influence the short- and long-term implant-related complication rate, as well as the possibility of achieving bony fusion, thus the therapeutic outcome.

The first application of finite element analysis (FEA) in biomechanics was published by Brekelmans et al. (1972). In the last decades, FEA contributed to the understanding of the spine, its components, and its behavior in healthy, diseased, or damaged conditions (Fagan et al., 2002), complementing the *in vitro* experiments. FEA has become a common research method in the field of *in silico* medicine (Viceconti et al., 2008).

To the best knowledge of the authors, there is no study comparing the 3 aforementioned OLIF implants with different bone material properties in the current literature. The aim of this study was to use FE analysis to evaluate the stability of different OLIF fusion constructs (BPS, LPS, and SSA) in normal and osteoporotic conditions. While a direct validation of the outputs of the models for this specific application was not the goal of this study, the present comparative computational approach enables to highlight the importance of bone material strength and stiffness reduction (ageing, metabolic bone diseases, etc.) in the surgeon’s decision-making process of choosing between different fixation options.

## Materials and Methods

### Generation of L2–L4 Lumbar Spine Bi-Segment Finite Element Model

A CT scan (Hitachi Presto, Hitachi Medical Corporation, Tokyo, Japan) of a 24-year-old patient’s lumbar spine was selected from a study of 270 patients who underwent different treatments due to lower back pain in our clinic (MySPINE, Project ID: 269909, Funded under: FP7-ICT). The imaging protocol was previously defined in the MySPINE project (Castro-Mateos et al., 2015), (Rijsbergen et al., 2018), and the images were reconstructed with a voxel size of 0.6 × 0.6 × 0.6 mm^3^. The L2–L3 and L3–L4 segments were not affected by any musculoskeletal pathology. The data were extracted from the hospital PACS in DICOM file format. To comply with the ethical approval of the patient data protection, de-identification of the DICOM data was performed using Clinical Trial Processor software (Radiological Society of North America, https://www.rsna.org/ctp.aspx) (Aryanto et al., 2015). In order to define the 3D geometry, we performed a segmentation procedure using Mimics image analysis software (Mimics Research, Mimics Innovation Suite v23.0, Materialise, Leuven, Belgium) via the Hounsfield thresholding algorithm and manual segmentation tools. To evaluate the accuracy of the segmentation process, we calculated the Dice Similarity Index (DSI) (Zou et al., 2004; Bharatha et al., 2001) based on two segmentation sessions of the same geometry.

From the segmented masks, a triangulated surface mesh was automatically generated in STL (Stereolithography) format. In 3-Matic (Mimics Research, Mimics Innovation Suite v21.0, Materialise, Leuven, Belgium) software, surface smoothing (iteration: 6, smoothing factor: 0.7, with shrinkage compensation) and uniform remeshing (target triangle edge length 0.6 mm, sharp edge preservation, sharp edge angle 60°) were applied on the 3D geometries.

**FIGURE 1:**
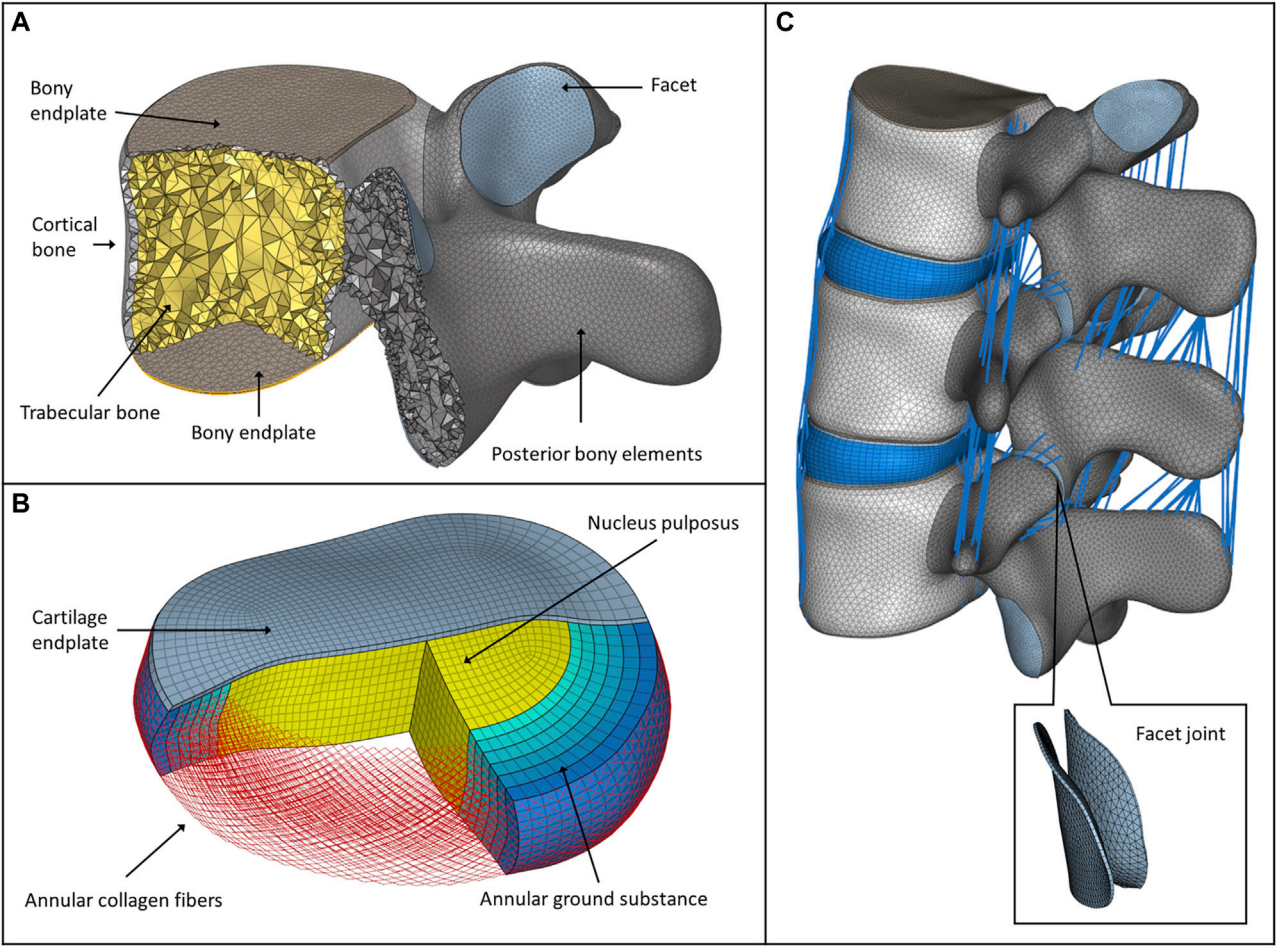
FE model of the intact L2–L4 spine bi-segment. **(A)** Model of the vertebral body, bony endplates, cortical shell, trabecular core, posterior elements, and articular facet. **(B)** Model of the intervertebral disc, nucleus pulposus, annular collagen fibers, and ground substance. **(C)** Intact L2–L4 lumbar spine bi-segment FE model, with facet joints and ligaments from a left posterior-lateral view.

In 3-Matic, the vertebrae were divided into posterior and anterior parts (Shirazi-adl et al., 1984). The anterior parts were divided into a cortical shell (thickness: 1 mm), vertebral bony endplates (thickness: 0.5 mm), and a cancellous core. Facet joints were modeled manually, with 0.25 mm cartilage height and a minimum 0.5 mm gap between the two facets (Dreischarf et al., 2014) (Figure 1A). The intersection-based non-manifold assembly was exported to Hypermesh software (Altair Engineering, Inc., Troy, Michigan, United States), and all of the surfaces were remeshed with a uniform triangulated surface mesh (target tringle edge length: 1 mm). From the resulting 3D surfaces, an adaptive tetrahedral volume mesh was generated, with the exception of the bony endplates, where pyramid elements were used (Table 1).

**TABLE 1:** Material properties and mesh type assigned to the FE model.

Material	Element type	Constitutive law	Young’s modulus (MPa)	Poisson ratio (-)	References	
Normal cortical bone	C3D4	Linear elastic	12,000	0.3	Shirazi-adl et al. (1984)
Osteoporotic cortical bone	C3D4	Linear elastic	8,040 (67% of normal)	0.3	(Polikeit et al., 2003), (Zhang et al., 2010), (Salvatore et al., 2018)
Normal cancellous bone	C3D4	Linear elastic	100	0.2	Shirazi-adl et al. (1984)
Osteoporotic cancellous bone	C3D4	Linear elastic	34 (34% of normal)	0.2	(Polikeit et al., 2003), (Zhang et al., 2010), (Salvatore et al., 2018)
Normal post. elements	C3D4	Linear elastic	3,500	0.25	Shirazi-Adl et al. (1986)
Osteoporotic post. elements	C3D4	Linear elastic	2,345 (67% of normal)	0.25	(Polikeit et al., 2003), (Zhang et al., 2010), (Salvatore et al., 2018)
Normal bony endplate	C3D4,C3D5, C3D8	Linear elastic	1,000	0.4	Silva et al. (1997)
Osteoporotic bony endplate	C3D4,C3D5, C3D8	Linear elastic	670 (67% of normal)	0.4	(Polikeit et al., 2003), (Zhang et al., 2010), (Salvatore et al., 2018)
Cartilaginous endplate	C3D8	Linear elastic	23.8	0.4	Lu et al. (1996)
Facet cartilage	C3D6	Neo-Hooke	C10 = 5.36; D1 = 0.04		Finley et al. (2018)
AF ground substance	C3D8	Neo-Hooke	C10 = 0.3448; D1 = 0.3		Rohlmann et al. (2009)
AF fibre	T3D2	Nonlinear stress–strain curve	Cross-sectional areas were calculated by a layer from volume fractions; 23% at the outermost layer to 5% at the innermost fibre layer		Shirazi-Adl et al. (1986), Lu et al. (1996)
Nucleus pulposus (NP)	C3D8H	Mooney–Rivlin	C10 = 0.12; C01 = 0.03; v = 0.4999		Schmidt et al. (2007)
Ligaments	SPRINGA	Nonlinear stress–strain curve (Table 2)	NA	NA	Rohlmann et al. (2006)
Bone graft	C3D4	Linear elastic	100	0.2	Akamaru et al. (2005)
PEEK cage	C3D4	Linear elastic	3,600	0.3	Zhang et al. (2018)
Titanium (screw, plate, and rod)	C3D4	Linear elastic	110,000	0.3	Zhang et al. (2018)

The annulus fibrosus (AF) and the nucleus pulposus (NP) defining the intervertebral disc were modelled manually according to the literature (Figure 1B) (Shirazi-Adl et al., 1986), (Schmidt et al., 2007). The NP accounted for 45% of the intervertebral volume and was moved in the posterior direction, so that the sagittal thickness of the posterior AF substance became 80% of the anterior AF (Shirazi-Adl et al., 1986). The fluid-like behavior of the NP was modeled using an isotropic, hyperelastic Mooney-Rivlin formulation (hexahedral mesh) (Schmidt et al., 2007). The AF consisted of 2 times 6 annulus fiber sets embedded into a hexahedral ground substance matrix of six layers with alternating orientations about ±30° to the mid-cross-sectional area of the disc (Lu et al., 1996). The fiber cross-sectional areas were calculated using the assumed collagen fiber volume fractions: 23% at the outermost layer, gradually decreasing to 5% at the innermost layer (Shirazi-Adl et al., 1986; Lu et al., 1996). The cartilaginous endplate thickness was set to 0.5 mm with hexahedral elements (Table 1), (Finley et al., 2018).

In total, seven ligaments were modeled as tension-only spring elements with non-linear material properties, namely, the ALL (anterior longitudinal ligament), PLL (posterior longitudinal ligament), LF (ligamentum flavum), ISL (interspinal ligament), SSL (supraspinal ligament), ITL (intertransverse ligament), and CL (capsular ligament) (Table 2). The attachment points, orientation and the element number of the ligaments were adopted from a previous study (MySPINE, Project ID: 269909, FP7-ICT), (Figure 1C **)**. The material properties were adopted from the literature (Rohlmann et al., 2006). The facet cartilage material was described by using a Neo-Hookean model, and a surface-to-surface contact without friction was set between the facet surfaces (Lu et al., 1996).

**TABLE 2:** Properties of the ligaments (Rohlmann et al., 2006).

Ligament	Stiffness (N/mm)	Strains between (%)	Stiffness (N/mm)	Strains between (%)	Stiffness (N/mm)	Strains higher than (%)
ALL	347	0–12.2	787	12.2–20.3	1864	20.3
PLL	29.5	0–11.1	61.7	11.1–23	236	23
LF	7.7	0–5.9	9.6	5.9–49	58.2	49
CL	36	0–25	159	25–30	384	30
ITL	1.4	0–13.9	1.5	13.9–20	14.7	20
SSL	2.5	0–20	5.3	20–25	34	25
ISL	0.3	0–18.2	1,8	18.2–23.3	10.7	23.3

### Cage, Implant Construct and Surgical FE Model Development

**FIGURE 2:**
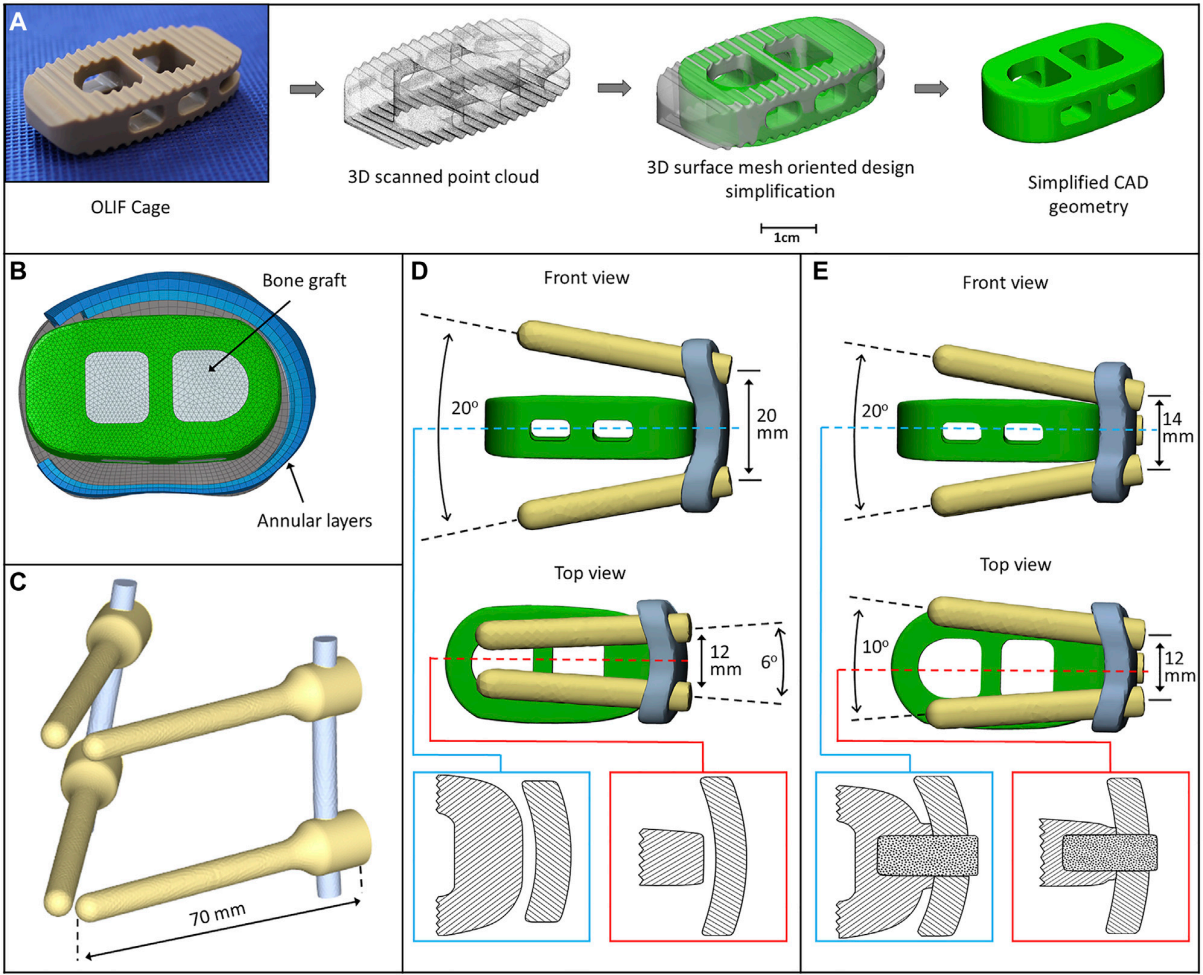
3D models of the implants. **(A)** Physical OLIF cage and virtually simplified CAD geometry obtained via 3D scanning (point cloud). The 3D surface mesh model oriented the design simplification process. **(B)** Position of the cage inside the intervertebral space. The internal space of the cage is filled with bone graft. **(C)** Bilateral pedicle screw fixation (BPS) model. **(D)** Cage model and lateral plate fixation system with screws (LPS). The cage is not connected to the plate (blue box: axial plane section, red box: sagittal plane section). **(E)** Cage connects to the plate with a screw (SSA) forming a self-anchoring mechanism (blue box: axial plane section, red box: sagittal plane section).

**FIGURE 3:**
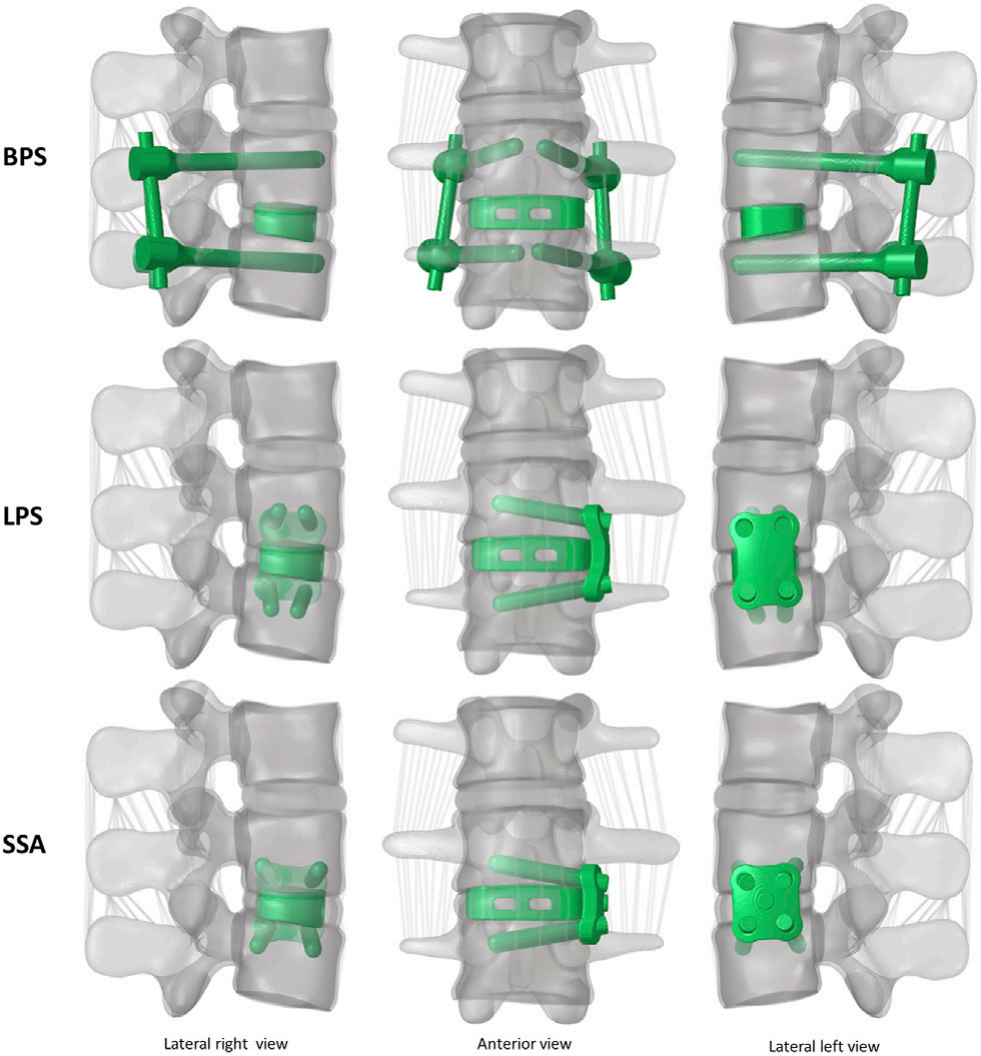
3D geometry of the bi-segment (L2–L4) model with the three investigated (OLIF) fixation constructs: OLIF cage with bilateral pedicle screws (BPS), OLIF cage with lateral plate system (LPS), OLIF cage with self-anchoring stand-alone system (SSA). Lateral left–right, and frontal view.

A PEEK (polyether-ether-ketone) OLIF cage (EMERALD™, Sanatmetal, Eger, Hungary, 45 mm × 22 mm × 12 mm, with 6⁰ lordosis) was scanned using a ScanBox 3D scanner (Smart Optics Sensortechnik GmbH, Bochum, Germany). The obtained point cloud was used to reconstruct the virtual 3D cage model using 3-Matic software. The model was exported in STL format to Autodesk Fusion 360 (Autodesk Inc., San Rafael, CA, United States) CAD (Computer Aided Design) software and served as a base for creating a simplified cage mesh (Figure 2A). The resulting geometry was used in all three (BPS, LPS, and SSA) FE models in the same central position. In order to simulate the surgical nucleotomy, the NP, 4 inner layers of the AF, and the cartilage endplates were removed from the investigated motion segment (L3–L4), and a window was created to insert the cage from the left side of the disc (Figure 2B)**.** For the BPS model, 4 identical simplified transpedicular screws (5.5 mm × 70 mm) were placed inside the L3 and L4 pedicles. The screwheads were connected using a 5.5-mm titanium rod (Figure 2C)**.** A lateral plate (32 mm × 23 mm × 4 mm) was designed to match the geometry of the L3 and L4 vertebrae with a coronal and an axial curvature for the LPS model, and 4 simplified lateral screws (40 mm × 5.5 mm) were inserted to fix the plate. There was no connection between the plate and the inserted cage (Figure 2D). For the SSA model, a smaller bicurved plate (26 mm × 23 mm × 4 mm) was anchored to the cage using a simplified screw (15 mm × 5.5 mm), and the 4 lateral screws were inserted at a different (diverging) axial angle compared to the LPS model (Figure 2E). “Tie constraint” was defined between bone–titanium, titanium–titanium, and PEEK–titanium contact surfaces to simulate rigid fixation, and in order to model the knurled surface of the PEEK cage, a 0.2-friction coefficient was set for the bony endplate–PEEK contact surfaces (Ambati et al., 2015). The material properties used in the intact and surgical models can be seen in Table 1. Osteoporotic bone mineral density was modeled by decreasing Young’s modulus of elasticity by a set amount (Table 1 **)**, (Polikeit et al., 2003), (Salvatore et al., 2018). Figure 3 presents the construction of OLIF models with various fixation options (BPS, LPS, SSA).

### Material Properties, Boundary and Loading Conditions, FE Model Validation

The intact L2–L4 and the six surgical bi-segment FE models (3 normal and 3 osteoporotic) were exported to Abaqus/CAEv11 (Dassault Systemes, Simulia Corp, Providence, RI, United States) software. Material properties and mesh types assigned to the FE models are summarized in Tables 1, 2. In order to validate the created L2–L4 intact model, a pure torque of 7.5 Nm was applied to the L2 vertebral body upper endplate in 3 general directions (flexion-extension, right/left bending, and right/left rotation), while the lower endplate of the L4 vertebra was fixed in place. The intact L2–L3 and L3–L4 segmental range of motions was compared to a cadaveric study (Ilharreborde et al., 2011). The lower endplate of the L4 vertebra was fixed in the case of the six surgical models as well. The simulations were conducted in two steps: first, a 150 N follower load was applied between the vertebral bodies; second, a pure 10 Nm torque was applied to the L2 vertebral body’s upper endplate in the three general directions used for the validation process.

## Results

### Model Validation

In order to evaluate the accuracy of the L2–L4 segmentation process, two investigators created the 3D geometries of the L2–L4 bony structures separately. The obtained DSI value for the vertebrae was 94%, indicating the high accuracy of the segmented models (Yao et al., 2016). The FE mesh quality was evaluated by defining the aspect ratio (AR) of the volume elements (Supplementary Table S1) and interpreted according to the literature (Burkhart et al., 2013), (Zhu et al., 2017).

**FIGURE 4:**
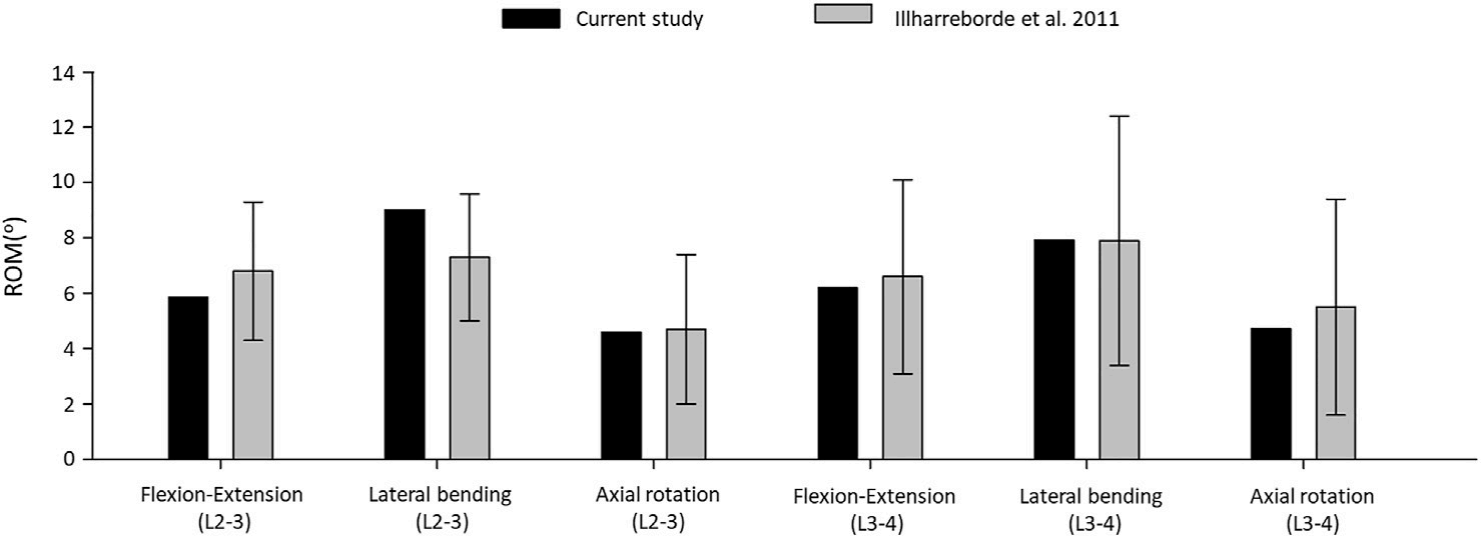
Comparison of the computed range of motions given by the intact L2–L4 bi-segmetal model with experimental results for 7.5-Nm pure moments.

The resulting ROMs were in accordance with the findings of a previous cadaveric study by Ilharreborde et al., 2011 (Ilharreborde et al., 2011), (Figure 4). The ROM of the L2–L3 segment in flexion–extension, lateral bending, and axial rotation was 5.86^°^, 9.01^°^, and 4.59^°^ respectively. In the cadaveric experiment, the corresponding ROM of the L2–L3 segment was 6.8° ± 2.5°, 7.3° ± 2.3°, and 4.7° ± 2.7°, respectively. For the L3–L4 segment, the ROM for flexion–extension, lateral bending, and axial rotation was 6.19°, 7.92°, 4.72°, respectively in our model and 6.6^°^ ± 3.5^°^, 7.9^°^ ± 4.5^°^, and 5.5^°^ ± 3.9^°^ for the cadaveric experiment, respectively.

The ROM comparison’s results suggested that the intact L2–L4 FE model in the present study was successfully constructed and could be used for further investigation.

### ROM, Displacement, and Cortical Endplate Stress Distribution

**FIGURE 5:**
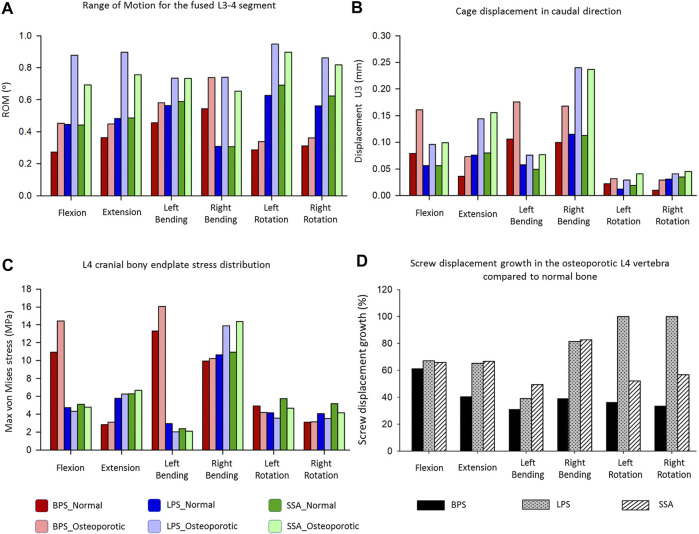
Results of the simulations extracted from the surgically reconstructed bi-segmental FEA model according to the six loading scenarios in normal and osteoporotic conditions. **(A)** Range of motion (ROM) values for the operated L3–L4 segment containing the investigated implant constructs (BPS: bilateral pedicle screw, LPS: lateral plate-screw, SSA: self-anchored stand-alone). **(B)** Cage displacement in the caudal direction (U3 in Abaqus). **(C)** Von Mises stress peaks on the L4 cranial bony endplate. **(D)** Measured L4 screw displacement increase (%) caused by osteoporotic bony conditions compared to L4 screw displacements inside the normal bone.

In order to compare the primary stabilizing properties of the 3 investigated implants, the ROMs of the virtually operated motion segments were compared. To evaluate the interaction between the inserted cage and the bony endplate below it, the cage’s caudal displacement and the endplate’s surface stress distribution were investigated. Additionally, the osteoporosis-induced increase in L4 screw displacement was studied to better understand which implant’s screws are the least affected by osteoporosis. A total of six surgical constructs were modeled and analyzed, corresponding to the BPS, LPS, and SSA fixation options with normal and osteoporotic bone material properties. The ROM of the surgical models under a combined load of 150 N follower load and 10 Nm torque is shown in Figure 5A. After the OLIF cage was inserted, the predicted ROM at the surgical level (L3–L4) decreased under all motion conditions compared with the intact model (Figures 4, 5A). Osteoporosis increased the ROM in all directions compared to the normal bone material property models. The highest impact caused by osteoporosis on the ROM occurred in the LPS fixation construct, where the ROM increased by 97.3% in flexion, 86.3% in extension, 30.14% in left bending, 140.26% in right bending, 50.96% in left rotation, and 53.38% in right rotation. The BPS provided the most stable primary fixation with low ROM values in normal and osteoporotic conditions with the exception of the right-bending scenario. The highest difference between the BPS and lateral plate systems (LPS, SSA) was found in the left- and right-side rotations. For normal bone, the difference in BPS vs LPS was 99% and BPS vs SSA was 119.73%, and for the porotic bone, BPS vs LPS was 158.94% and BPS vs SSA was 145.49%.

Osteoporosis increased the cage’s displacement in the caudal direction (U3) for all of the fixation constructs (Figure 5B). The highest increase in displacement was found in right bending for the LPS (from 0.115 to 0.24 mm, 109%) and for the SSA (from 0.113 to 0.237 mm, 110%) fixation. With the exception for flexion and left bending, the BPS fixation had lower displacement values both for normal and osteoporotic conditions compared to the LPS and SSA fixation. Overall, the cage displacement values were similar for the SSA and LPS fixation.

**FIGURE 6:**
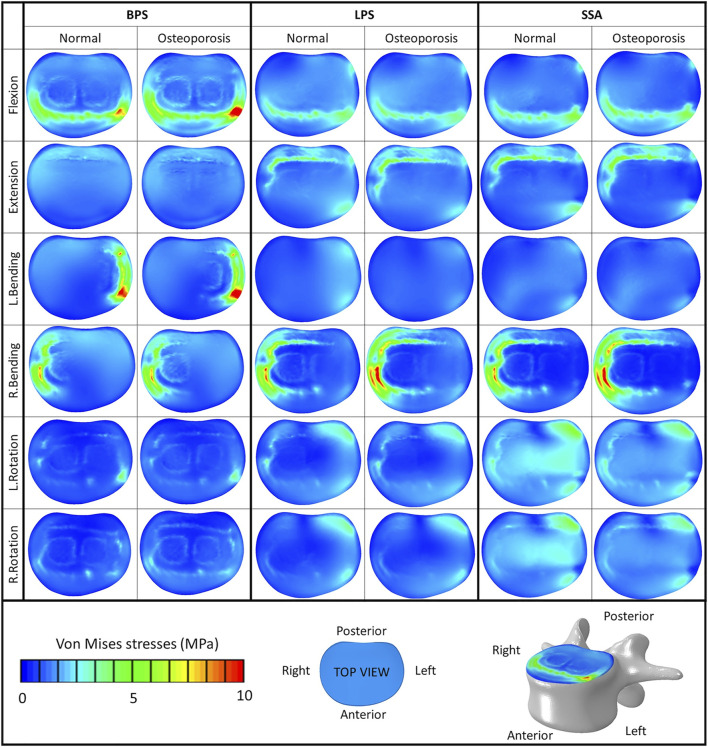
Von Mises stress distribution on the cranial bony endplate of the L4 vertebra, with various fixation options (BPS: bilateral pedicle screw, LPS: lateral plate-screw, SSA: self-anchored stand-alone) in normal and osteoporotic conditions under six loading scenarios. Color bar (blue/green/red), scale (0–10 MPa), top view.

The von Mises stress peaks on the L4 upper cortical endplate are shown in Figure 5C. Compared to the normal bone, the stress peaks increased in the osteoporotic models for extension, right bending, and right rotation. In flexion and left bending, the stress peaks for the BPS model were much higher compared to the other models (LPS, SSA) regardless of the bone material properties (Von Mises stress peaks for BPS were 10.92 and 13.31 MPa for flexion and left bending in normal bone, respectively, and 14.43 and 16.06 MPa in osteoporotic condition, respectively). To investigate this phenomenon, the von Mises stress distribution on the L4 upper cortical endplates was visualized using contour plots (Figure 6). This showed that the exceeding von Mises stress peaks in flexion and left bending for the BPS models are stress concentrations at the place of the fenestration made on the AF and the OLIF cage border.

The screw displacement was measured by highlighting the screw tips inside the L4 vertebra in the 3 constructs. The distance between two points were measured: Point 1: screw tip location before applying the forces. Point 2: screw tip location after the last “step” (“step” is a basic concept in Abaqus FE solver software) (Manual, 2020) of the simulation in a direction, and the result was the average of the 2 values (there were always 2 screws inside the L4 vertebra).

Osteoporosis increased the screw displacement in the L4 vertebra in all motion conditions compared to the normal bone models (Figure 5D). The highest increase was found in the case of LPS fixation for left (100%) and right (100%) rotation. The impact of osteoporosis on the BPS fixation’s screw displacement was lower in all of the six modeled motions, compared to the other two implants (screw displacement increase in the BPS model for flexion: 61.38%, extension: 40.38%, left bending: 31%, right bending: 39%, left rotation: 36.32%, and right rotation: 33.48%).

## Discussion

In the past decade, due to the advancement in minimally invasive spinal fusion technologies (Yue et al., 2010), the OLIF procedure has emerged, and it has been used ever more often by spine surgeons. The advantages of the OLIF surgical technique include the preservation of the posterior structures of the lumbar spine, reduced blood loss, and shorter hospital stay (Phan et al., 2016). Despite the fact that OLIF has been successfully adopted in the clinical environment, the risks of cage subsidence and screw loosening are possible postoperative complications related to this technique (Quillo-Olvera et al., 2018). Biomechanical failure of the stabilization construct (cage subsidence and loosening of the screws) is a multifactorial phenomenon (damage to endplates during preparation, overdistraction, cage design, etc.) (Quillo-Olvera et al., 2018). Bone quality as well as the biomechanical stability of the whole fusion construct can play a significant role in the development of this complication, possibly influencing the short- and long-term therapeutic outcome. The present study aimed to investigate the effect of bone quality on the stability of a fused segment with the aid of FEA models in 3 different fixation options.

First, an intact L2–L4 bi-segment FE model was developed and validated by comparing the ROMs (ante-retroflexion, lateral flexion, and rotation) under a pure 7.5 Nm torque to the findings of a previous cadaveric study by Ilharreborde et al. (Ilharreborde et al., 2011). The adequate validation results (Figure 4) allowed us to take a step further and modify the FE model to establish different OLIF construct models: BPS, LPS, and SSA with normal and osteoporotic bone material properties (Figure 3).

The ROM of the surgical models under a combined load of 150 N follower load and 10 Nm torque (Figure 5A) showed different behaviors based on the fixation type and bone material properties (normal/osteoporotic). The BPS fixation provided the most stable primary fixation in both normal and osteoporotic conditions. These findings are in accordance with the study by Guo et al. (Guo et al., 2020) who used a L3–L5 bi-segment FE model to evaluate OLIF constructs with various fixation options under the same combined loading of 150 N follower load and 10 Nm torque. They applied normal bone material properties and found similar results for the ROMs and also found that the BPS fixation provides the highest stability compared to lateral-only fixations.

To our knowledge, the first study to apply FE models to establish an osteoporotic spine model (L1–S1) to research the single-segment (L3–L4) biomechanical stability of OLIF with different fixation methods was recently published by Song et al. (Song et al., 2021). They found a similar trend in their results that the BPS fixation provides a more stable fixation than lateral plates in normal and osteoporotic conditions despite the differences between their (boundary conditions: axial compressive preload of 400 N, and torsional moment of 10 Nm) and our models. Song’s lateral plate fixation design concept differed from our model (the lateral plate was fixed to the vertebral body with 2 screws in their model, while 4 screws were used in our constructs), and his investigation did not include the SSA OLIF cage concept. Based on our results, osteoporosis increased the ROM in all motion conditions compared to the normal bone material property models. The highest impact caused by osteoporosis on the ROM occurred in the LPS fixation construct. The highest difference between the BPS and lateral plate systems (LPS, SSA) was found in rotational movements.

Osteoporosis increased the cage displacement in the caudal direction (U3 in Abaqus) for all of the fixation options (Figure 5B). Overall, the cage displacement values were similar for the SSA and LPS systems. The highest increase in displacement was found in right bending for the LPS and SSA implants. With the exception of flexion, and left bending BPS fixation had lower displacement values both for normal and osteoporotic conditions. Parallel to the caudal displacement, the opposite side of the cages can move in the cranial direction (Supplementary Figure S1). The complex mechanism of subsidence involving the upper and lower endplates supported by radiologic findings is still widely investigated (Quillo-Olvera et al., 2018).

Compared to normal bone, in the osteoporotic models we have measured increased values for the von Mises stress peaks on the L4 upper cortical endplate (Figure 5C **)** for extension, right bending, and right rotation. In flexion and left bending, the BPS model’s peek stress values were much higher compared to the other models (LPS, SSA) regardless of the bone material properties. Stress concentrations at the place of the AF fenestration and the OLIF cage border occurred in the BPS model in flexion and left bending (Figure 6). In the other loading scenarios, higher stresses can be observed on the endplate surface for the LPS and SSA models compared to the BPS model. Song’s study found that on the investigated L4 endplate, the stress increases with osteoporosis, but it is lower for the BPS implants compared to lateral plate fixation (Song et al., 2021).

Osteoporosis increased the screw displacement in the L4 vertebra in all motion conditions compared to normal bone models (Figure 5D). The highest increase was found in the case of LPS fixation for left (100%) and right (100%) rotation. The impact of osteoporosis on the BPS fixation screw displacement was lower in all of the six modeled motions compared to the other fixations.

The results of this study highlight that the possible advantages of the LPS and SSA fixations (e.g., lower operation time and invasiveness due to the lack of the posterior–percutaneous–fixation steps) could be hindered in osteoporotic patients. In osteoporotic patients, the BPS fixation provides a more stable fixation than the LPS and SSA fixation, which is important to avoid mechanical complications and provide optimal therapeutic outcome.

Although the FE analysis has many advantages over *in vitro* experiments, it has limitations as well, for example, its inability to “perfectly” mimic the human tissue mechanics. In order to simulate certain biomechanical processes inside the human body, simplifications need to be performed due to the limitations of *in silico* software. Osteoporotic and normal bone qualities are not uniformly distributed within the human skeleton. There can be vertebrae and regions inside the vertebrae that are more affected by osteoporosis and can lead to weaker spots. This line of thought leads to an infinite amount of bone material property distribution models, so we have chosen the path of creating a uniform bone material model for our investigations. The osteoporosis FE model was constructed by decreasing the elastic modulus of the normal uniform cortical and cancellous bone by a certain proportion. However, in the literature, more complex approaches are described to model osteoporosis, by integrating micro-level trabecular structural mechanics (McDonald et al., 2010). With ageing, degenerative changes can occur in the spine not only affecting the vertebral bone material properties but also the geometry (exp. stabilizing osteophytes) (Margulies et al., 1996) and the internal structure of the intervertebral disc as well. Therefore, in an osteoporotic model, the non-surgically treated discs and bony structures should be altered accordingly.

In this study, the osteoporotic model had the same bony geometry and intervertebral disc material properties as the normal bony model.

The developed model investigated the primary stability of the constructs right after the surgery, not taking into consideration the expected fusion process because long-term bony fusion is often the desired result of an adequately chosen implant and correctly executed surgery.

## Conclusion

Bilateral pedicle screw (BPS) and rod fixation provided superior primary biomechanical stability for OLIF cages, compared to self-anchored stand-alone (SSA) or lateral plate-screw fixated (LPS) cages in both normal and osteoporotic conditions. Osteoporosis amplified the difference between the stability of the bilateral pedicle screw fixation and the two other investigated fixation methods. Clinically, in the case of decreased bone quality (primary or secondary osteoporosis), the surgeon has to take into consideration the limits of the SSA and LPS fixations, despite the advantage that there is no need for a second step in the surgery by turning the patient to the prone position to perform the percutaneous pedicle screw fixation. This study highlights the need for further investigation (experimental and clinical trials) to adjust the indication of the fixation methods in OLIF to the patient’s bone quality.

## Data Availability

The original contributions presented in the study are included in the article/Supplementary Material; further inquiries can be directed to the corresponding author.
