# Supplementary material for: Stability Evaluation of Different Oblique Lumbar Interbody Fusion Constructs in Normal and Osteoporotic Condition – A Finite Element Based Study
Source: Front Bioeng Biotechnol. 2021 Nov 5;9:749914. doi: 10.3389/fbioe.2021.749914 (PMC8602101; doi:10.3389/fbioe.2021.749914)
Supplement: Supplementary file 1 [file DataSheet1.pdf]

Supplementary Materials

Stability Evaluation of Different Oblique Lumbar Interbody Fusion Constructs in Normal and Osteoporotic Condition – a Finite Element Based Study

Ferenc Bereczki[1,2], Mate Turbucz[1,2], Rita Kiss[3], Peter Endre Eltes[1,3*], Aron Lazary[3,6*]

1. In Silico Biomechanics Laboratory, National Center for Spinal Disorders, Budapest, Hungary
2. School of PhD Studies, Semmelweis University, Budapest
3. Department of Mechatronics, Optics and Mechanical Engineering Informatics, Budapest University of Technology and Economics, Budapest, Hungary
4. Department of Spine Surgery, Semmelweis University, Budapest, Hungary

*authors contributed equally to the work

Ferenc Bereczki
National Center for Spinal Disorders, Királyhágó St. 1-3, Budapest 1126, Hungary
Tel.:(36) 1-887-7900, Fax.: (36) 1-887-7987 Email address: ferenc.bereczki@bhc.hu

Mate Turbucz
National Center for Spinal Disorders, Királyhágó St. 1-3, Budapest 1126, Hungary
Tel.:(36) 1-887-7900, Fax.: (36) 1-887-7987, Email address: turbucz95@gmail.com

Rita Kiss
Department of Mechatronics, Optics and Mechanical Engineering Informatics, Budapest University of Technology and Economics, Budapest 1111, Bertalan Lajos St. 4-6. Building D 407, Budapest, Hungary
Tel.:(36) 1-463-1738, Email: rita.kissmogi@bme.hu

Peter Endre Eltes, corresponding author
National Center for Spinal Disorders, Királyhágó St. 1-3, Budapest 1126, Hungary
Tel.:(36) 1-887-7900, Fax.: (36) 1-887-7987, Email address: eltespeter@yahoo.com

Aron Lazary,
National Center for Spinal Disorders, Királyhágó St. 1-3, Budapest 1126, Hungary
Tel.:(36) 1-887-7900, Fax.: (36) 1-887-7987, Email address: aron.lazary@bhc.hu

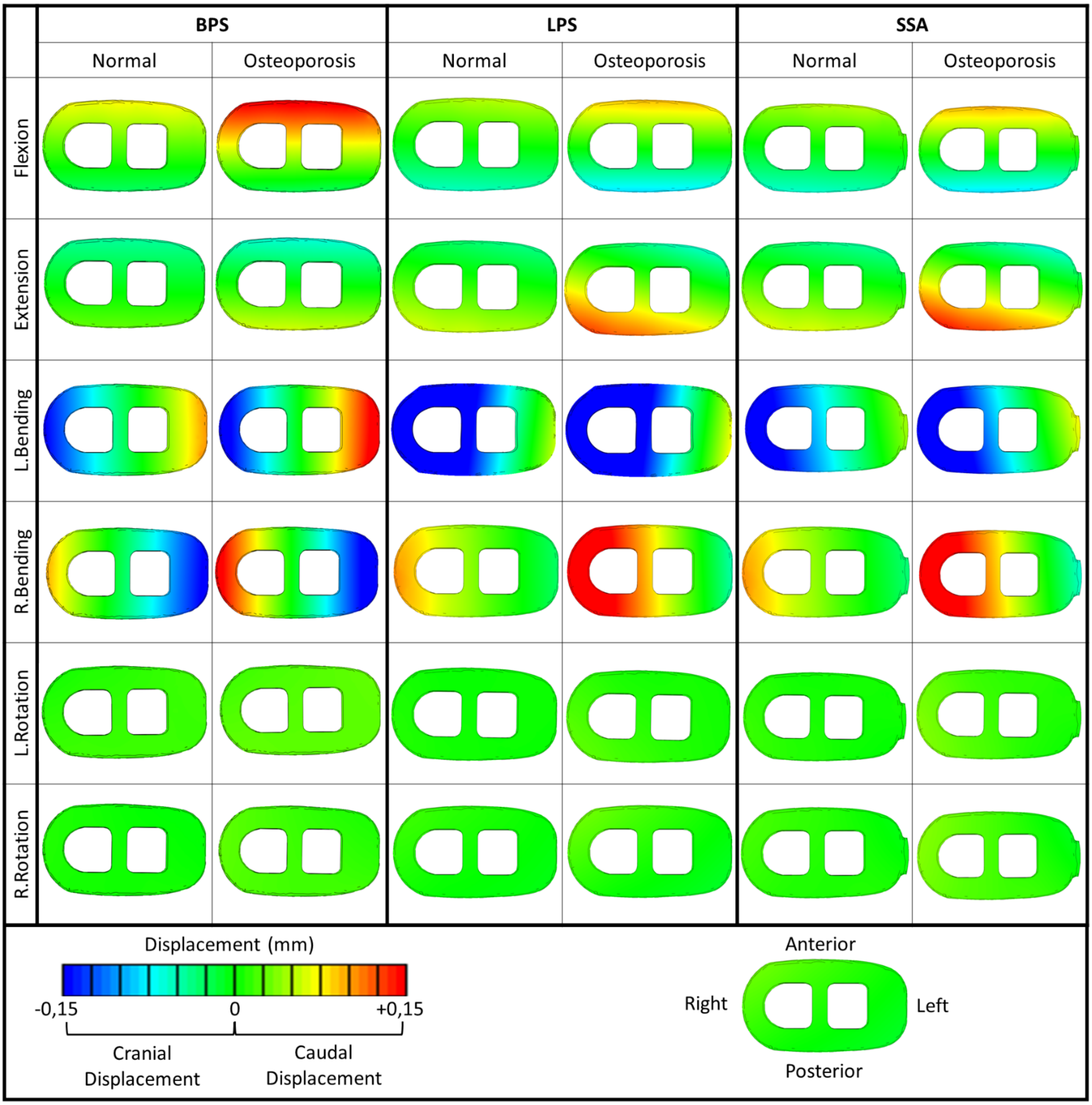

Supplementary Figure 1. Colour map of the cage displacements (U3) in the cranio-caudal direction in the three OLIF models with various fixation options (BPS: bilateral pedicle screw, LPS: lateral plate-screw, SSA: self-anchored standalone) in normal and osteoporotic bone material property condition. Displacement is represented by the colorbar (Blue/Green/Red), scale: -0.15–0.15 mm, bottom view.

Supplementary Table 1. The calculated Aspect Ratio (AR) of the volume elements building up the finite element mesh. According to the literature: 1<AR<3: acceptable; 3<AR<10: treated with caution, AR>10: treated with alarm [1].

Level	Parts	Element number	1<AR<3	%	3<AR<10	%	10<AR	%
**L2**	Cortical bone	15182	15149	99,78	33	0,22	0	0
	Trabecular bone	47612	47602	99,98	10	0,02	0	0
	Bony endplates	28225	24909	88,25	3316	11,75	0	0
	Post. bony elements	379801	378421	99,64	1380	0,36	0	0
	Facet joints	35317	35317	100,00	0	0,00	0	0
**L3**	Cortical bone	18169	18143	99,86	26	0,14	0	0
	Trabecular bone	47264	47264	100,00	0	0,00	0	0
	Post. bony elements	20147	18444	91,55	1703	8,45	0	0
	Post. elements	490942	490526	99,92	416	0,08	0	0
	Facet joints	46430	46430	100,00	0	0,00	0	0
**L4**	Cortical bone	16444	16373	99,57	71	0,43	0	0
	Trabecular bone	54631	54587	99,92	44	0,08	0	0
	Bony endplates	30845	28583	92,67	2262	7,33	0	0
	Post. bony elements	564319	562549	99,69	1770	0,31	0	0
	Facet joints	53032	53032	100,00	0	0,00	0	0
**L2-3**	Cartilage endplates	3542	3538	99,89	4	0,11	0	0
	Nucleus	11470	11427	99,63	43	0,37	0	0
	Annulus	6240	6240	100,00	0	0,00	0	0
**L3-4**	Cartilage endplates	3744	3612	96,47	132	3,53	0	0
	Nucleus	12600	12552	99,62	48	0,38	0	0
	Annulus	6120	6120	100,00	0	0,00	0	0
**Implant**								
**CAGE**		123722	123612	99,91	110	0,09	0	0
**GRAFT**		71856	71758	99,86	98	0,14	0	0
**PLATE_SA**		48794	48708	99,82	86	0,18	0	0
**PLATE_ST**		59321	59126	99,67	195	0,33	0	0
**POST_SCREW**		151603	151581	99,99	22	0,01	0	0

References:

[1] Burkhart TA, Andrews DM, Dunning CE. Finite element modeling mesh quality, energy balance and validation methods: A review with recommendations associated with the modeling of bone tissue. J Biomech 2013;46:1477–88. https://doi.org/10.1016/j.jbiomech.2013.03.022.
